# Regulation of limited N-terminal proteolysis of APE1 in tumor via acetylation and its role in cell proliferation

**DOI:** 10.18632/oncotarget.8026

**Published:** 2016-03-10

**Authors:** Kishor K. Bhakat, Shiladitya Sengupta, Victor F. Adeniyi, Shrabasti Roychoudhury, Somsubhra Nath, Larry J. Bellot, Dan Feng, Anil K. Mantha, Mala Sinha, Suimin Qiu, Bruce A. Luxon

**Affiliations:** ^1^ Department of Genetics, Cell Biology and Anatomy, University of Nebraska Medical Center, Omaha, NE-68198, USA; ^2^ Fred and Pamela Buffet Cancer Center, University of Nebraska Medical Center, Omaha, NE-68198, USA; ^3^ Department of Biochemistry and Molecular Biology, University of Texas Medical Branch, Galveston, TX-77555, USA; ^4^ Current Affiliation: Department of Radiation Oncology, Houston Methodist Research Institute, Houston, TX-77030, USA; ^5^ Bioinformatics Program, University of Texas Medical Branch, Galveston, TX-77555, USA; ^6^ Department of Pathology, University of Texas Medical Branch, Galveston, TX-77555, USA; ^7^ Current Affiliation: Molecular Biology Research and Diagnostic Laboratory, Saroj Gupta Cancer Centre and Research Institute (SGCC & RI), Kolkata-63, India

**Keywords:** APE1, acetylation, base excision repair, proteolysis, proliferation

## Abstract

Mammalian apurinic/apyrimidinic (AP) endonuclease 1 (APE1), a ubiquitous and multifunctional protein, plays an essential role in the repair of both endogenous and drug-induced DNA damages in the genome. Unlike its *E.coli* counterpart Xth, mammalian APE1 has a unique N-terminal domain and possesses both DNA damage repair and transcriptional regulatory functions. Although the overexpression of APE1 in diverse cancer types and the association of APE1 expression with chemotherapy resistance and poor prognosis are well documented, the cellular and molecular mechanisms that alter APE1 functions during tumorigenesis are largely unknown. Here, we show the presence of full-length APE1 and N-terminal truncated isoforms of APE1 in tumor tissue samples of various cancer types. However, primary tumor tissue has higher levels of acetylated APE1 (AcAPE1) as well as full-length APE1 compared to adjacent non-tumor tissue. We found that APE1 is proteolytically cleaved by an unknown serine protease at its N-terminus following residue lysine (Lys) Lys6 and/or Lys7 and after Lys27 and Lys31 or Lys32. Acetylation of these Lys residues in APE1 prevents this proteolysis. The N-terminal domain of APE1 and its acetylation are required for modulation of the expression of hundreds of genes. Importantly, we found that AcAPE1 is essential for sustained cell proliferation. Together, our study demonstrates that increased acetylation levels of APE1 in tumor cells inhibit the limited N-terminal proteolysis of APE1 and thereby maintain the functions of APE1 to promote tumor cells' sustained proliferation and survival.

## INTRODUCTION

Apurinic/apyrimidinic (AP) endonuclease 1 (APE1) is the primary enzyme in mammals responsible for the repair of spontaneously generated abasic sites in the genome [1, 2]. APE1 also plays a central role in the repair of both endogenous and environmentally-induced oxidative and drug-induced DNA damages in the genome via the base excision repair (BER) pathway [1–3]. In addition to its DNA repair function, APE1 also activates the DNA-binding activity of many stress-inducible transcription factors (TFs) including c-Jun, P53 and NF-kβ through its redox activator (Ref-1) function [4–7]. Finally, APE1 has a third and distinct function; it also functions as a direct transcriptional co-activator or co-repressor of many genes involved in cell growth and chemotherapeutic drug resistance [7–12].

APE1 is essential for embryonic development [13] as well as for cell viability and/or proliferation in culture [14, 15]. Human APE1 has an N-terminal disordered domain (1-42 amino acid; aa) and possesses both DNA repair and transcriptional regulatory activities [16]. The N-terminal domain contains the nuclear localization signal [17] and multiple Lys residues that can be modified by acetylation and ubiquitination [18–20]. The C-terminal portion (aa 61-318) of APE1 is essential for DNA repair function (AP-endonuclease activity) [21]. Previously, we reported that Lys6 and Lys7 in the N-terminal domain of APE1 can be acetylated (AcAPE1) by the histone acetyltransferase (HAT) p300 in cultured cells and this acetylation modulates the transcriptional co-regulatory activity of APE1 [9, 11, 18]. Other Lys residues (Lys27, 31, 32 and 35) in the N-terminal domain of APE1 are also found to be modified by acetylation in HeLa cells and mutation of these residues can modulate the DNA damage repair activity of APE1 [19].

Overexpression of APE1 in tumor cell lines and various cancer tissues including lung, ovarian, colon, glioma, head and neck, breast, and prostate have been observed [22–24]. APE1 levels, as well as its altered subcellular localization, have been associated with tumor promotion and progression as well as poor prognosis and lack of responsiveness to chemotherapy [24–26]. Knockdown or impairment of the redox or DNA repair function of APE1 by small molecules inhibitors inhibits tumor cell proliferation and sensitizes cells to a variety of chemotherapeutic agents [24, 27–29]. Given the elevated levels of APE1 in tumor cell lines and cancer tissues of diverse origins, this enzyme has emerged as an important therapeutic target [24, 26].

Overexpression of APE1 in a variety of cancers as well as its association with chemotherapy resistance and poor prognosis has been well documented. Little is known about how the different functions of APE1 may be regulated via alteration of post-translational modifications during tumorigenesis. In this study, we show that the post-translational regulation of APE1 in tumor tissue is distinct from that observed in cultured cells. Specifically, we observed that primary tumor tissues have higher levels of AcAPE1 as well as full-length APE1 compared to adjacent non-tumor tissues. We characterized that the N-terminal domain (1-33 amino acids) of APE1 is cleaved by a limited proteolysis in both tumor and adjacent non-tumor tissue. However, in tumor tissue, the N-terminal limited proteolysis of APE1 is reduced by the enhanced acetylation of multiple Lys residues in this domain. Modulation of APE1 or AcAPE1 levels in tumor cells alters expression of hundreds of genes and APE1 acetylation is essential for sustained cell proliferation.

## RESULTS

### Presence of full-length APE1, N-terminal truncated APE1 isoforms and elevated levels of AcAPE1 in tumor samples

**Figure 1:**
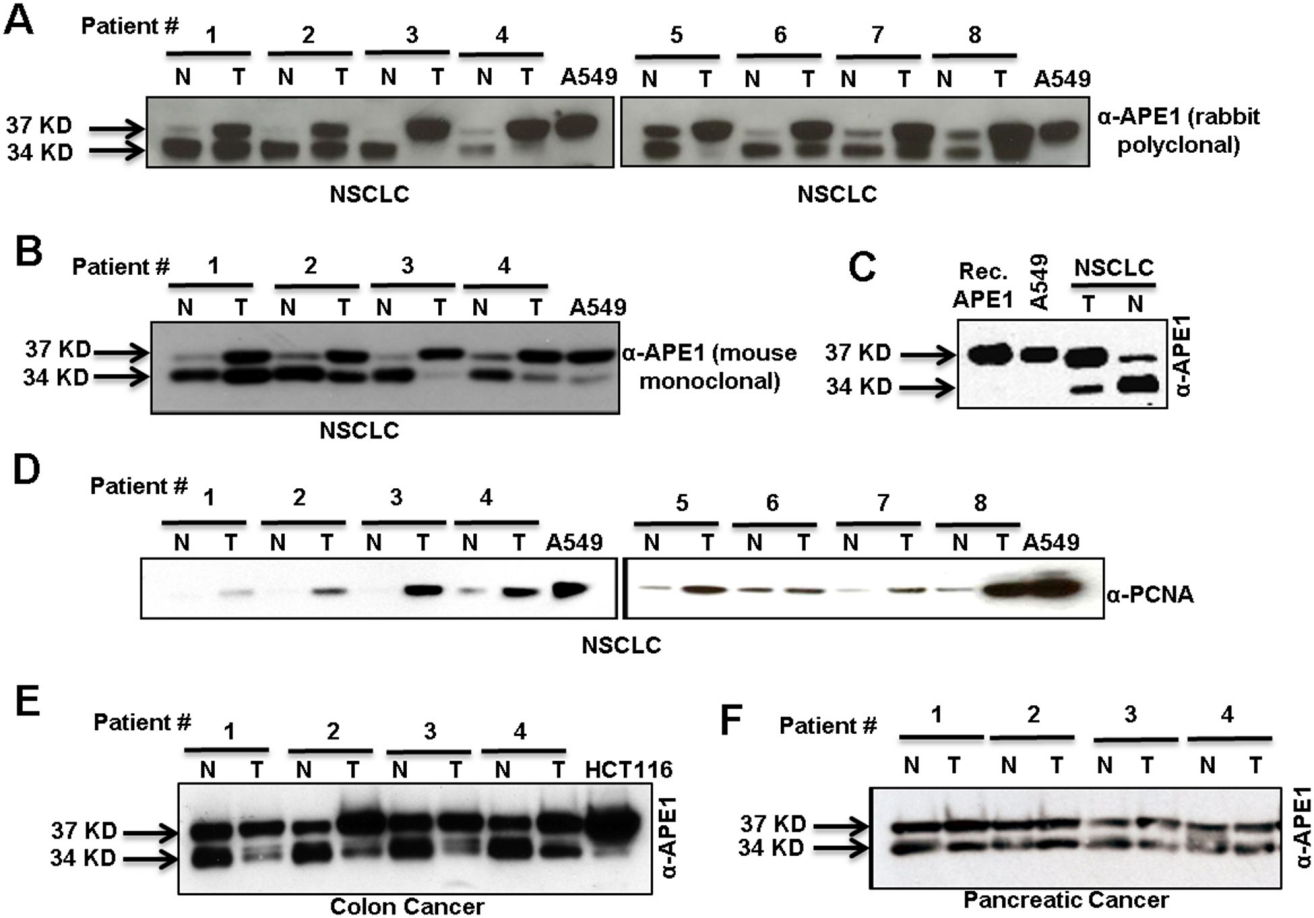
Two isoforms of APE1 in tumor and adjacent non-tumor tissue samples of diverse origins **A & B.**Western blot analysis of tumor-adjacent non-tumor (N) and tumor (T) tissue lysates from NSCLC cancer patients with (A) rabbit polyclonal α-APE1 Ab or (B) mouse monoclonal α-APE1 Ab. **C.** Western blot analysis of recombinant (Rec.) APE1, A549 cell lysate and tissue lysates from tumor and adjacent-non-tumor regions of the same NSCLC patient using α-APE1 Ab. **D.** Western blot analysis of tumor and non-tumor tissue extracts from NSCLC patients with α-PCNA Ab. **E & F.** Western blot analysis of tumor and adjacent non-tumor tissue extracts of patients with colon (E) and pancreatic (F) cancer with α-APE1 Ab.

We compared APE1 levels in non-small cell lung carcinoma (NSCLC) tissue samples to the adjacent non-tumor tissue from patients. Western blot analysis using anti-APE1 antibodies (Ab) revealed increased levels of APE1 in the tumor tissue compared to adjacent non-tumor tissue (Figures 1A & 1B). Notably, the predominant APE1 band observed in all non-tumor tissue had faster mobility compared to the 37 kD band corresponding to APE1 present in cultured lung adenocarcinoma A549 cells and full-length (FL) recombinant (Rec.) APE1 (Figures 1A&1C). By contrast, all tumor tissue demonstrated a prominent FL APE1 and the faster-migrating APE1 bands (Figures 1A & 1B). In contrast, NEIL1, another DNA BER enzyme, was present as a single band in both tumor and adjacent normal tissues from NSCLC patients (Figure S1). As expected, we observed a significant increase in PCNA level, a marker for cell proliferation in tumor extracts compared to non-tumor (Figure 1D). The two APE1 isoforms and their differential abundance patterns were also observed in tumor versus adjacent non-tumor tissue in colon and pancreatic cancer patients (Figure 1E & 1F). Together, these data suggest the presence of two isoforms of APE1 in a variety of cancer types.

**Figure 2:**
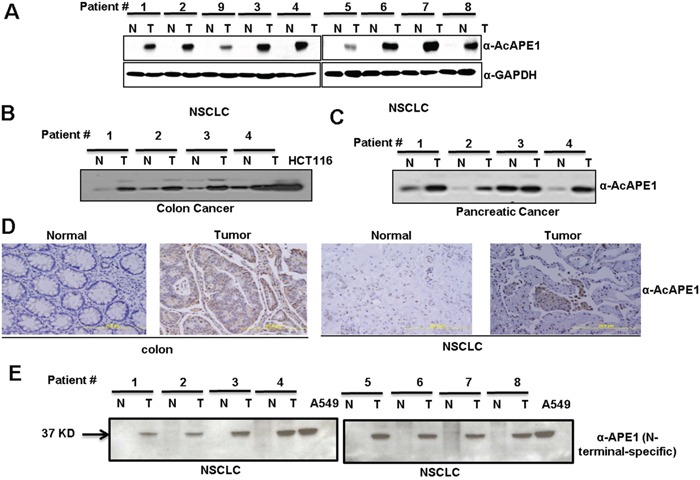
Elevated levels of AcAPE1 in tumor tissue **A.** Western blot analysis of tumor-adjacent non-tumor (N) and tumor (T) tissue lysates from NSCLC cancer patients with α-AcAPE1 Ab. GAPDH was used as a loading control. **B & C.** Western blot analysis of tumor and tumor adjacent-non-tumor tissues of patients with colon (B) and pancreatic (C) cancer with α-AcAPE1 Ab. **D.** Representative immunohistochemical staining of AcAPE1 in paraffin-embedded colon cancer and NSCLC tissue sections. **E.** Western blot analysis of non-tumor (N) and tumor (T) tissue lysates from NSCLC cancer patients used in Figure 1A with an N-terminal-specific Ab of APE1.

Next, we investigated the acetylation status of APE1 in NSCLC tissue utilizing our previously generated AcAPE1-specific Ab [9, 11]. Specifically, this AcAPE1-specific Ab was generated using the N-terminal peptide containing 1-13 amino acids (aa) with acetylated Lys at position 6 [9]. We have previously shown that this Ab is highly specific in recognition of AcAPE1 species acetylated at Lys6 position and does not cross react with 50-fold excess of unmodified APE1 [9]. Furthermore, this antibody recognized ectopic WT APE1 but was unable to detect non-acetylable K6R/K7R APE1 mutant protein [7, 30]. Using AcAPE1 Ab we found that the levels of AcAPE1 in tumor-adjacent non-tumor tissue were below the level of detection. However, AcAPE1 levels were significantly increased in all tumor tissue (Figure 2A). Likewise, we observed a significant increase in AcAPE1 levels in colon and pancreatic cancer tissue relative to non-tumor controls (Figure 2B & 2C). Immunohistochemical analysis confirmed increased nuclear AcAPE1 staining in tumor compared to non-tumor tissue (Figure 2D). The near complete absence of AcAPE1 in NSCLC-adjacent non-tumor tissue (Figure 2A) raises the possibility that APE1 may be truncated at the N-terminus. In fact, we observed that in tumor-adjacent non-tumor tissue extract, APE1 was not detected by the Ab specific to the APE1 N-terminal peptide (residues 1-14), but a significant APE1 band was present in corresponding tumor tissue extracts (Figures 2E & S2). As expected, the faster-migrating band in adjacent-non-tumor tissue was observed when the same immunoblots were probed with an Ab raised against full-length APE1 (Figure 1A). The absence of APE1 splice variants in either tumor or non-tumor tissue as confirmed by RT-PCR (data not shown) eliminates the possibility that alternative RNA splicing produces the N-terminally deleted APE1 isoform. These collective data strongly suggest that both full-length APE1 and its N-terminal truncated isoform are present in tumor and tumor-adjacent non-tumor tissue and that elevated levels of AcAPE1 are also present in tumor tissue compared to adjacent-nontumor tissue.

### Serine protease(s) cleaves the N-terminus of APE1

**Figure 3:**
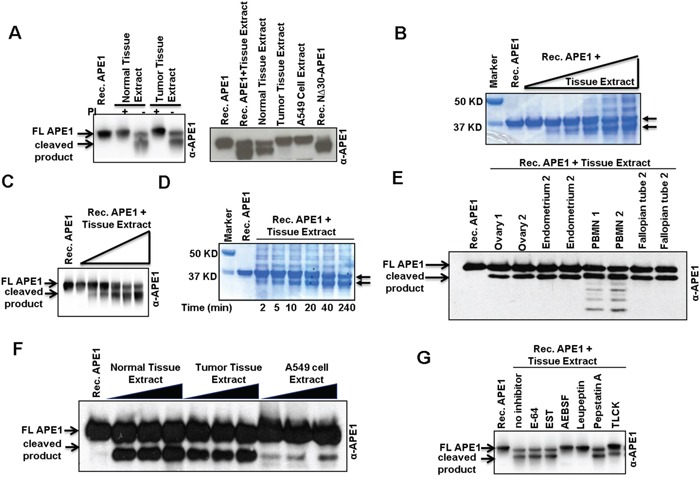
N-terminal limited proteolysis of APE1 by a putative serine protease(s) and its presence in tissue extracts **A.** Western blot analysisof Recombinant (Rec.) APE1 after incubation with NSCLC or tumor-adjacent non-tumor tissue extracts isolated in the presence (+) or absence (−) of protease inhibitors (PI). **B & C.** Rec. APE1 was incubated with increasing amounts of tumor-adjacent non-tumor tissue extract (isolated in the absence of PI) from a NSCLC patient, separated by SDS-PAGE and (B) visualized by Coomassie Blue staining or (C) immunoblotted with α-APE1 Ab. **D.** Time-dependent cleavage of Rec. APE1 with constant amount of the tissue extract. Arrow denotes truncated APE1 isoforms. **E.** Rec. APE1 was incubated with normal tissue extracts from healthy person (isolated in the absence of PI), and then immuno-blotted with α-APE1 Ab. **F.** Cleavage of Rec. APE1 with NSCLC tissue and A549 cell extracts (isolated in the absence of PI). **G.** Effect of different classes of PI on cleavage activity of normal tissue extracts on Rec. APE1. FL: full length.

To test if a protease cleaves the N-terminal segment of APE1, recombinant APE1 protein was incubated with tumor or tumor-adjacent non-tumor tissue extract. Two truncated forms of APE1 were produced in the absence of protease inhibitors (Figure 3A, left panel), and were identical to the APE1 isoforms observed in the patient tissue samples (Figure 3A, right panel). This data indicates that both tumor and tumor-adjacent non-tumor tissue extracts have proteolytic-processing activity that cleaves recombinant APE1 in vitro in the absence, but not in the presence, of protease inhibitor cocktails (Figure 3A). Moreover, by incubating a constant amount of recombinant APE1 with increasing amounts of non-tumor tissue extracts, the shift from FL to two truncated forms of APE1 occurred in a dose- and time- dependent manner indicating a limited proteolysis of FL APE1 (Figures 3B, 3C & 3D). Extracts isolated from tumor-adjacent tissues of patients with colon, pancreas, ovarian and lung cancer (Figure S3), as well as resected normal tissues from the ovary, endometrium, fallopian tubes and peripheral blood mononuclear (PBMN) cells of healthy persons (Figure 3E), also demonstrated APE1-specific cleavage activity in this *in vitro* assay. Extracts from cultured A549 cells also showed APE1 cleavage activity, albeit to a much lesser extent (Figure 3F). Like APE1, histone H3 has positively charged unstructured N-terminal (1-35 aa) domain. DNA glycosylase NEIL1 has a C-terminal (289-389 aa) unstructured domain [31, 32]. However, the absence of cleavage of either recombinant Histone H3 or NEIL1 (Figure S4) in this in vitro assay indicates that the protease(s) responsible for APE1 cleavage in the tissue extracts does not cleave all proteins that have unstructured N- or C-terminal domain. Using specific inhibitors of various classes of proteases, we identified the APE1-cleaving protease(s) to be serine protease(s) as both reversible serine protease inhibitor AEBSF and irreversible trypsin-like serine protease inhibitor leupeptine completely prevented APE1's proteolysis (Figure 3G). By contrast, cysteine-specific inhibitor E64, or aspartic acid protease inhibitor pepstatin A did not prevent the proteolysis of APE1. Thus, the proteolysis of the N-terminal domain of APE1 is mediated by a trypsin-like serine protease(s).

### Putative serine protease(s) cleaves APE1 after Lys6 or Lys7, Lys27 and Lys31 or 32

**Figure 4:**
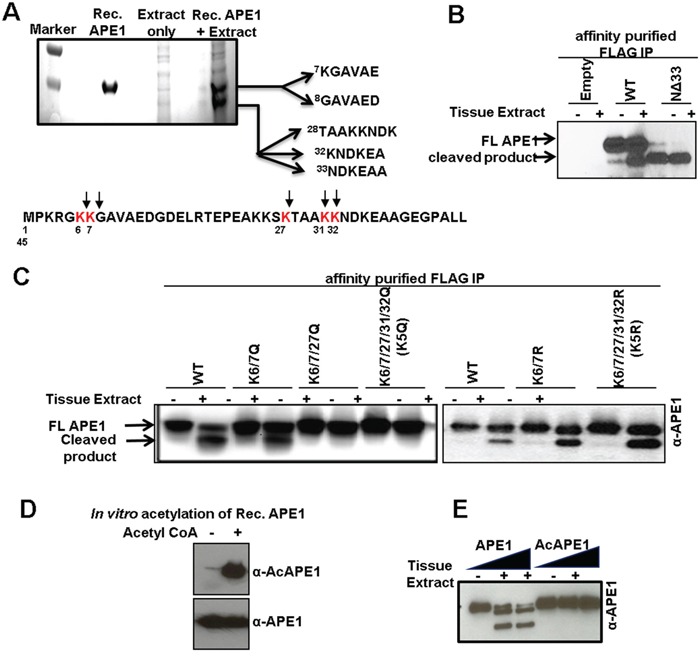
Identification of protease-mediated cleavage sites in APE1 and inhibition of this proteolysis by acetylation **A.** Coomassie Blue-stained SDS-PAGE for N-terminal sequencing shows two cleaved APE1 isoforms; the N-terminal 1-45 aa sequence of APE1 showing cleavage sites (bottom panel). **B.** Western blot analysis of FLAG-immuno-affinity purified FLAG-tagged WT APE1, N-terminal 33 aa deleted (NΔ33) mutant incubated ± tissue extract in the absence of PI. **C.** Western blot analysis of FLAG-immuno-affinity purified FLAG-tagged WT APE1, or site specific mutants incubated ± tissue extract in the absence of PI. **D & E.** Rec. APE1 was *in vitro* acetylated followed by Western blot analysis with α-AcAPE1, α-APE1 Abs (D) to confirm acetylation, and (E) incubated with normal tissue extract followed by Western blot analysis.

To determine the nature of the truncated N-terminal forms of APE1, we isolated the two APE1 isoforms generated after proteolysis by SDS-PAGE and transferred them to a nylon membrane for N-terminal sequencing by Edman degradation. Cleavage following residue Lys6 and/or Lys7 generated the higher molecular weight proteolytic product (top band), the lower molecular weight proteolytic product resulted from cleavage of the N-terminal segment following Lys27, Lys31 and/or Lys32 (Figure 4A). Thus the lower molecular weight band corresponds to a mixture of un-resolved APE1 bands cleaved after residues Lys27 and Lys31 or Lys32. Taken together these data indicate that a currently unknown protease(s) cleaves APE1 in between Lys6 and 7 or after Lys7 and also after Lys27 and Lys31 or 32; thus generating primarily two N-terminally truncated isoforms of APE1 (NΔ7 and NΔ27 or NΔ32; Figures 3C & 3D). Incubation of immunoprecipitated FLAG-tagged WT APE1 but not an N-terminal 33 aa deletion mutant (NΔ33), generated truncated isoforms of APE1 confirming that the primary cleavage sites of the protease(s) are located within N-terminal domain 33 aa residues (Figure 4B). Mutation of all five Lys sites (Lys6/7/27/31/32) to glutamine (K5Q; Figure 4C, left panel), but not to arginine (K5R; Figure 4C, right panel) completely prevented proteolysis of APE1, confirming these Lys residues as proteolytic cleavage sites in APE1.

### Acetylation of Lys residues in N-terminal domain of APE1 prevents proteolytic-cleavage in tumor tissue

Despite the presence of the APE1-specific proteolytic-processing activity in both tumor and adjacent-non-tumor tissue extracts, we observed significant levels of FL APE1 in tumor tissue. This raises the question of what specifically protects APE1 molecules from N-terminal cleavage in tumor cells. One or multiple mechanisms may be involved in regulation of limited proteolysis of APE1. Previously, we discovered that the N-terminal Lys6/7/27/31/32 residues of APE1 can be reversibly acetylated in cells [18, 19]. Because these residues are also susceptible to cleavage by APE1-specific serine protease(s) (Figure 4A) we postulated that acetylation of these residues may prevent proteolytic cleavage of APE1 in tumor tissue. Consistent with this hypothesis, we found that the protease(s) present in tissue extracts were unable to cleave recombinant acetylated APE1 (Figure 4D and 4E). Thus it is possible that acetylation of the N-terminal Lys residues protects APE1 from limited proteolysis and the increased acetylation level of APE1 in tumor tissues is one of the mechanism by which tumor cells prevent N-terminal proteolysis of APE1.

### AcAPE1 level changes during cell cycle and AcAPE1 is exclusively present in the nucleus

**Figure 5:**
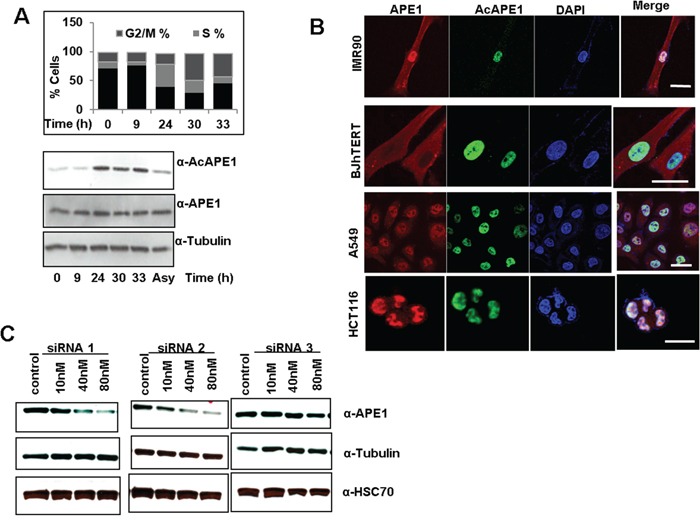
Cell-cycle-dependent APE1 acetylation and sub-cellular localization of AcAPE1 **A.** Single parameter propidium iodide-staining based cell cycle/FACS analysis (upper panel) and Western blot analysis (bottom panel) of extracts isolated at indicated time points from serum-starved (72 hrs) BJ-hTERT cells followed by serum supplementation. **B.** Confocal microscopy images of primary lung fibroblast IMR-90, hTERT-transformed diploid BJ cells, lung adenocarcinoma A549 and colon cancer HCT116 cells fixed with paraformaldehyde and immunostained using α-APE1, α-AcAPE1 Abs and counter stained using DAPI. **C.** Western blot analysis of APE1 protein levels from A549 cells at 72 hrs after transfection with various doses of three independent APE1-specific siRNAs or control siRNA. Western analysis of cell extracts with α-tubulin (middle) and HSC70 (bottom) Abs.

The fundamental difference between terminally differentiated cells and cancer cells is that the later cells are continuously proliferating and/or progression of the cell cycle. To examine whether the levels of AcAPE1 changes with the cell cycle, we first determined the doubling time of hTERT-transformed normal diploid BJ fibroblasts cells (Figure S5). To assess AcAPE1 level in hTERT-BJ cells we utilized synchronization to G_0_/G1 via serum starvation for 72 hrs, followed by releasing the cells by serum supplementation. We observed that AcAPE1 levels increased as cells progressed from the G1 to S phase and remained elevated throughout mitosis (Figure 5A). The total level of APE1 remained unchanged. We investigated the sub-cellular localization of AcAPE1 using the AcAPE1 Ab. Confocal microscopy data indicated that AcAPE1 staining was strictly nuclear, whereas APE1 was observed both in nucleus and cytoplasm in primary lung fibroblasts IMR-90 cells and hTERT-BJ fibroblast (Figure 5B). Furthermore, exclusive localization of AcAPE1 in the nucleus was also observed in lung adenocarcinoma A549 and colon cancer HCT116 cells (Figure 5B).

### Modulation of endogenous APE1 or its acetylation level alters the gene expression profile

**Figure 6:**
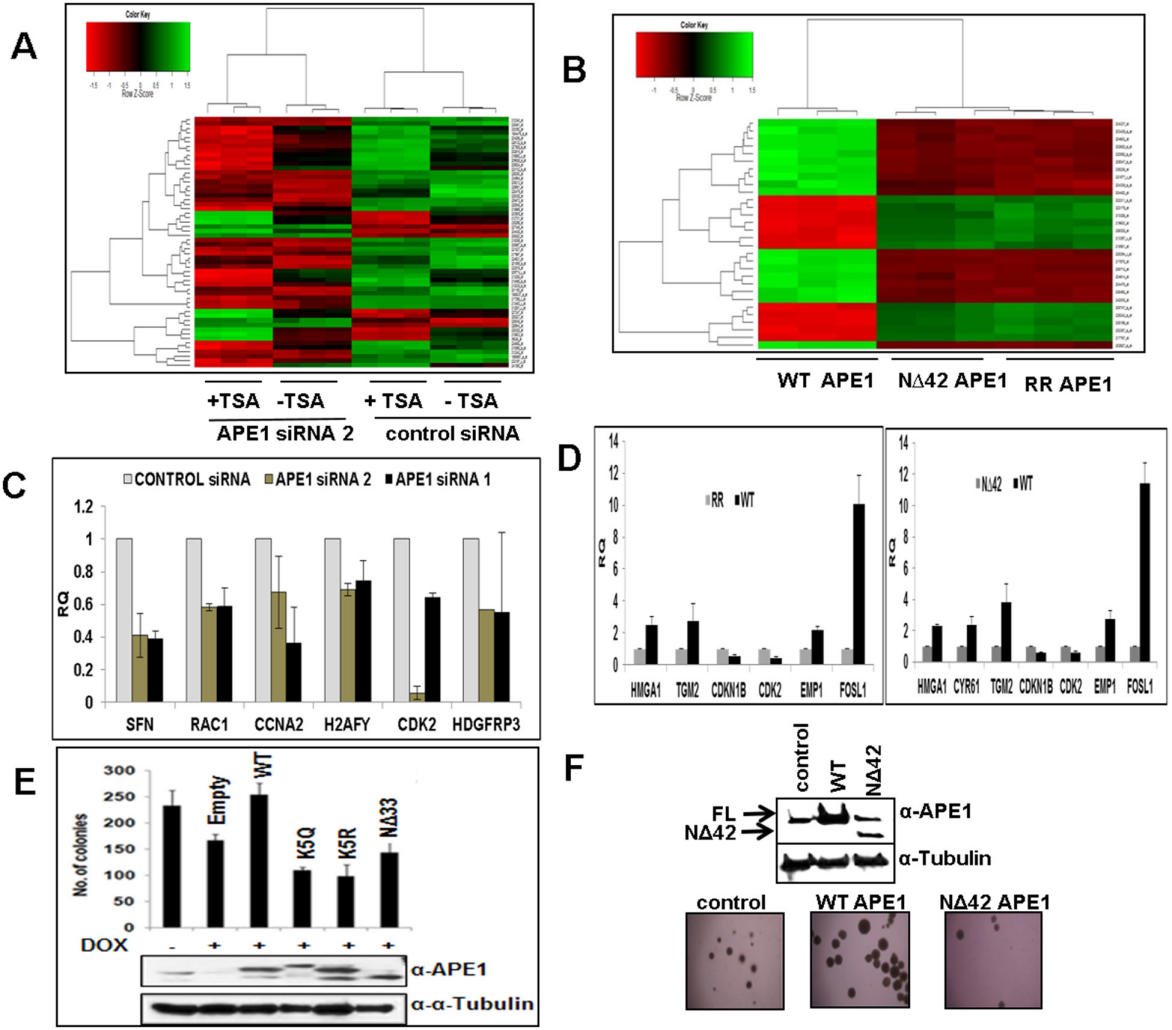
APE1 N-terminal domain or its acetylation regulates expression of multiple genes and essential for sustained cell proliferation **A.** Heat Maps (Bioconductor/R; Limma) generated from micro-array based GeneChip analysis shows differential gene expression profile of control siRNA, APE1-siRNA 2 (80nM), TSA-treated control and TSA-treated APE1 siRNA-transfected A549 cells (p =0.001). Three independent biological replicates of each group are represented. Color intensities correspond to relative signal levels as measures of mRNA expression on a logarithmic scale. **B.** Real Time RT-PCR analysis showing steady-state mRNA level in relative quantitation (RQ) with respect to HPRT1 of randomly selected cell proliferation-related APE1/AcAPE1-regulated genes in control vs. APE1-siRNA1 and APE1-siRNA2 knockdown A549 cells. Error bars denote SD (N=2-3). **C.** Heat Maps (Bioconductor/R; Limma) generated from micro-array based GeneChip analysis shows differential gene expression profile of ectopic WT, NΔ42 and K6R/K7R (RR) mutant APE1-expressing BEAS-2B cells (p = 0.00001). Three independent biological replicates of each group are represented. **D.** Real Time RT-PCR analysis showing steady-state mRNA level in relative quantitation (RQ) with respect to HPRT1 of randomly selected cell proliferation-related APE1/AcAPE1-regulated genes in ectopic WT vs. RR APE1 and WT vs. NΔ42 APE1 expressing BEAS-2B cells. Error bars denote SD (N=2-3) **E.** Endogenous APE1 was downregulated with doxycycline (Dox) treatment in HEK293T cell line stably expressing APE1 siRNA from a doxycycline-inducible promoter HEK293T^APE1siRNA^. Approximately 500 cells were plated on 60-mm dishes and allowed to grow for two weeks until visible colonies appear. The number of viable colonies was counted in cells in which endogenous APE1 was downregulated and in which FLAG-tagged WT APE1 or acetylation defective (mutations of Lys6,7,27,31&32 to non-acetylable arginine (K5R) or glutamine (K5Q) or N-terminal deletion mutants was ectopically expressed(APE1 levels in cell extracts are shown in inset) Error bars denote SD (N=3). **F.** Soft-agar assay for anchorage-independent growth of BEAS-2B cells with stably expressing ectopic WT or N-terminal 42 aa deleted (NΔ42) mutant APE1.

We showed previously that acetylation of APE1 regulates its transcriptional regulatory functions and modulates expression of multiple genes [7, 9, 11, 30]. Given its elevated levels in various tumors, we hypothesized that APE1 and its acetylation regulate expression of diverse sets of gene and promotes cell proliferation. To explore this possibility, we first examined the effect of various doses of three different APE1-sepcific siRNA oligonucleotides on downregulation of endogenous APE1 level in A549 cells. Figure 5C shows that while APE1-specific siRNA1 and siRNA2 were highly effective in decreasing the APE1 protein level in a dose-dependent manner, siRNA3 had not much effect. Furthermore, consistent with previous reports by us and others [9, 27, 33], we observed a significant (>80%) reduction in the APE1 protein level at a 80 nM dose of siRNA, without having any off-target effects as evidenced by the absence of changes in the α-tubulin and HSC70 protein levels (Figure 5C). Using Affymetrix HGU133 Plus 2.0 array, we compared the gene expression profile of A549 control cells to APE1-depleted A549 cells as well as A549 cells in which the AcAPE1 levels were further increased by treatment with the histone deacetylase inhibitor, trichostatin A (TSA; Figure 6A & S6). Bioconductor/R Limma-based data analysis identified modulation of 743 unique genes in APE1-depleted cells compared to control (fold change ≥ 1.25; list of genes in Supplementary Table 1). Further analysis of TSA-treated control versus TSA-treated APE1-depletion identified up- or down-regulation of 732 unique genes (fold change ≥ 1.25; genes list in Supplementary Table 2). A representative Heat Map (Figure 6A) revealed the set of genes, affected either by modulating endogenous APE1 level (control vs. APE1-siRNA2) or its acetylation (TSA-treated control vs. TSA-treated APE1 siRNA), that are likely to be specifically regulated by acetylation of APE1. Using Real Time RT-PCR analysis we validated the expression of some of these genes involved in cell proliferation: *SFN, RAC1, CCNA2, H2AFY, CDK2, HDGFRP3* in control vs. APE1- knockdown (K/D) A549 cells with both APE1-siRNA1 as well as APE1-siRNA2 (Figure 6C). The similar expression patterns of these genes after downregulation of APE1 level by two independent APE1-specifc siRNA confirms that these genes are regulated by APE1 (Figure 6C). Ingenuity Systems based pathway analysis showed a significant proportion of genes involved in cell survival and proliferation pathways are deregulated by elevated AcAPE1 in A549 cells (Figure S7) with a strong linkage to cancer (Figure S8).

To further confirm that the N-terminal domain of APE1 and its acetylation are involved in modulating gene expression, we examined the effect of ectopic WT, non-acetylable K6R/K7R (RR) or N-terminal 42 aa deleted (NΔ42) APE1 mutants. We used immortalized bronchial epithelial BEAS-2B cells which express low levels of APE1 for ectopically expressing these mutants (levels are shown by Western blot analysis in Figure S9). A representative Heat Map (Figure 6B) revealed that many of the same gene were up- or down-regulated in RR and NΔ42 expressing cells relative to the WT control ( list of genes shown in Supplementary Tables 3 and 4), thus confirming the role of APE1 N-terminal region in gene regulation through acetylable Lys residues. A Venn diagram analysis (Figure S10) also showed that a significant proportion of the genes that were up- or down-regulated in the WT versus NΔ42 comparison were similarly differentially expressed in the WT versus RR analyses comparison. We further validated the expression of some of these genes involved in cell proliferation such as *HMGA1, TGM2, CDKN1B, CDK2, EMP1* and *FOSL1* in BEAS-2B cells expressing WT vs. RR (Figure 6D, left panel) or WT vs. NΔ42 APE1 (Figure 6D, right panel). Ingenuity Systems based pathway analysis displayed that significant proportion of genes involved in cell growth and survival pathways are affected in the absence of N-terminal domain of APE1 or its acetylation (Figure S11).

### APE1 acetylation is required for cell proliferation

The requirement of APE1 acetylation for regulation of expression of diverse sets of gene in multiple pathways raise the possibility that elevated levels of AcAPE1 in tumor may be essential for sustained proliferation of tumor cells. We sought to determine the requirement of the acetylation of APE1 for cell proliferation. We used our previously generated HEK293T^APE1siRNA^ cells stably expressing APE1 siRNA under an inducible doxycycline promoter. Inducible-downregulation of endogenous APE1 in HEK293T^APE1siRNA^ cells by doxycycline (Dox)-treatment [11], significantly decreased the number of viable cells. This effect could be rescued by ectopically expressing WT APE1 but not with the non-acetylable K5R, K5Q and NΔ33 mutants (Figure 6E). Our data show that acetylation/deacetylation cycles of Lys residues the N-terminal domain of APE1 also play an essential role in cell growth and proliferation because mutation of the five acetylable Lys (Lys 6,7,27,31 &32) residues to either non-acetylable Arginine (K5R) or acetylation mimic K5Q mutant affects the cell growth and proliferation. Furthermore, using a soft agar assay, we found that stable expression of WT APE1, but not the N-terminal deleted APE1 mutant enhances the anchorage-independent growth of BEAS-2B cells (Figure 6F). Together, these data suggest that not only the N-terminal domain but also acetylation/deacetylation of Lys residues in this domain plays an essential role in cell growth and proliferation.

## DISCUSSION

Over the last 20 years, APE1 expression and its subcellular localization has been primarily examined in fixed, paraffin-embedded, tissue-sections from diverse cancer types and matched controls using immunohistochemical techniques [24, 26]. By contrast, in the present study, using immunoblot analysis of cancer tissue lysates we discovered that post-translational regulation of APE1 in tumor tissue is distinct from that observed in fixed tissue section and cultured cells. A consensus exists among several studies that all (tumor and transformed) cell lines contain predominantly full-length APE1 [7, 9, 11, 34, 35]. In contrast, we observed that in tumor and adjacent non-tumor tissue APE1 is proteolytically cleaved at its N-terminus by a currently unknown serine protease(s). Enhanced acetylation of APE1 in tumor cells inhibits this proteolysis and our data show that the acetylation of N-terminal domain of APE1 is involved in modulating the expression of genes involved in sustained cell proliferation and/or survival. Thus, increased APE1 acetylation levels in tumor cells and the resulting inhibition of N-terminal limited proteolysis of APE1 represent a novel mechanism by which cancer cells maintain APE1 functions and thereby sustain expression of genes associated with cell cycle progression and survival.

Our novel findings of limited N-terminal proteolysis of APE1 and the existence of two APE1 isoforms in cancer were unexpected because this phenomenon was not reported previously. The failure to observe these isoforms previously is likely due to the fact that unlike our current study that employed immunoblot analysis, most of the earlier studies utilized immunohistochemical analysis of tissue-sections. Using immunoblot analysis of various cancer tissue samples, we have established the presence both full-length APE1 and its truncated two isoforms in cancer. However, unlike tumor and adjacent non-tumor tissue extracts from cancer patients, all cultured cell lines have predominantly full-length APE1 [7, 34, 35]. This raises the question regarding what signals and/or conditions trigger the APE1 N-terminal proteolysis in tumor and in adjacent non-tumor tissue. We postulate that multiple signals are essential for activation of APE1-proteolysis in tumor and adjacent non-tumor tissue. The tumor microenvironment, which is characterized by acute/chronic hypoxia, low extracellular pH levels, elevated oxidative stress and altered interaction of tumor cells with stromal cells, likely contributes to the activation of the APE1 proteolysis by the protease [36]. In addition, the presence of the truncated isoforms of APE1 in adjacent non-tumor tissues suggests that the surrounding non-malignant tissue is likely to be influenced by the adjacent tumor. It is also now clear that interplays between tumor cells and the microenvironment are complex and exert a profound influence on adjacent-normal tissue [36]. Cleavage of the APE1 N-terminus by a protease under certain conditions is not unprecedented. Indeed, previous studies have shown that the APE1 N-terminal domain (1-33 aa) is cleaved after induction of DNA damage in HL60 cells [37]. Similarly, inhibition of protein synthesis or mitochondrial electron chain transportation was shown to induce the cleavage of N-terminal 33 amino acids of APE1 in HeLa cells [34]. It has also been shown that APE1 is targeted and cleaved at Lys 31 by Granzyme A (GzmA), a highly abundant serine protease found in cytotoxic granules of T-lymphocytes during cytotoxic T-cell-mediated apoptosis [38]. Still, the APE1-specific serine protease described in this study is unlikely to be GzmA given its presence in many tissues. Our data show that serine protease cleaves APE1 at or after multiple Lys residues in the N-terminal domain of APE1 that undergo posttranslational modification by acetylation in cells. However, the protease was unable to cleave the N-terminal domain of APE1 when it is acetylated. We should point out that in addition to these five acetylable Lys residues the N-terminal domain (1-42 aa) of APE 1 contains multiple Lys and arginine residues including Lys 3, Lys 24, Lys 25, Lys 35, Arg 4 and Arg18. However, our in vitro cleavage assay of recombinant APE1 by tissue extracts revealed that the protease cleaves specifically at or after acetylable Lys 6, 7 and Lys27, 31 and 32. Because these Lys residues can be acetylated in cells and APE1 acetylation is enhanced in S-phase, higher levels of AcAPE1 in tumor cells is consistent with the predominant presence of full-length APE1 in tumor tissue compared to adjacent non-tumor tissue. Moreover, earlier studies demonstrated that APE1 acetylation level increased after oxidative and /or genotoxic stress or under inflammatory condition in cells [30, 39]. Thus we propose that alteration of APE1 acetylation level in tumor cells in vivo could be one of the mechanisms by which proteolysis of APE1 is prevented under malignant condition. However, we cannot eliminate the possibility of involvement of other mechanisms including some other posttranslational modifications of APE1 and/or the protease itself in regulation of APE1 cleavage. Our lab is currently working on this issue.

Our study raises one fundamental question about the role of N-terminal domain of APE1 and its acetylation in cells. It is intriguing to note that unlike its *E. coli* counterpart Xth, mammalian APE1 is unique in that it has a disordered N-terminal 42 aa extension and possesses both DNA repair as well as transcription regulatory functions [40]. We showed earlier that all cells irrespective whether they are primary normal or transformed or tumor have AcAPE1 [7, 9]. This suggests that AcAPE1 performs some important functions in cells. Consistent with this idea, previous studies by our group and others have shown that acetylation/deacetylation of the Lys residues of APE1 modulates both its transcriptional regulatory function and DNA repair function via maintaining association and dissociation of APE1 with the partner proteins [9, 19, 41, 42]. Studies by our group and others have shown that the N-terminal 33 aa of APE1 is required for interaction with its many interacting partners proteins [8-11, 35], and APE1 acetylation modulates their interactions [9, 11, 19]. We showed earlier that APE1 acetylation enhances its interaction with YB-1 and could promote chemotherapeutic drug-resistance by modulating expression of the key drug efflux transporter protein MDR1 [9, 11]. Moreover, previous studies have revealed the association of APE1 with RNA Pol II complex and with TFs including p53, YB-1, estrogen receptor and coactivators like p300 on the promoter bound complexes of many genes [8, 11, 43]. Consistent with this, our current study show that deletion of APE1 N-terminal domain or modulation of its acetylation in multiple tumor cell lines affects expression of hundreds of genes essential for cell survival and proliferation. Although validation of these results with patients' primary tumor samples is important, however, our gene expression data with A549 as well as immortalized lung epithelial BEAS-2B cell line revealed significant overlap of the genes that are affected after APE1 downregulation or in the absence of N-terminal of APE1 or its acetylation. Moreover, our IPA analysis revealed that the APE1 N-terminal domain and its acetylation regulate expression of genes that are linked to sustained cell proliferation and growth. Thus our data strongly suggest that APE1 and its acetylation play an important role in activation of a subset of proliferation-specific as well as housekeeping genes for maintaining sustained proliferation and survival. Because the N-terminal domain and the acetylable Lys residues of APE1 modulates both its transcriptional regulatory and DNA damage repair functions, it is expected that acetylation/deacetylation cycles of Lys residues the N-terminal domain of APE1 also play an essential role in cell growth and proliferation. Supporting this, we showed earlier that all cultured tumor cell lines have higher AcAPE1 levels compared to non-tumorigenic cell lines [7, 9]. In this study, we discovered an additional role of acetylation of APE1 in tumor cells in regulating the limited N-terminal proteolysis of APE1 under malignant condition. Thus, we propose that under malignant condition in vivo cell-cycle-dependent increased APE1 acetylation in proliferating cancer cells not only inhibits proteolytic cleavage of the N-terminal domain but also facilitates its transcriptional regulatory function to promote cell proliferation and survival [7, 19, 41]. In summary, our study demonstrates that the N-terminal domain of APE1 is cleaved by an unknown serine protease in tumor. Enhanced APE1 acetylation in tumor cells prevents this proteolysis and thereby maintains transcriptional regulatory function of APE1 and promote cell proliferation and survival.

## MATERIALS AND METHODS

### Patients tissue samples, extraction of tissue lysates and Western blot analysis

The resected frozen tissues from non-small cell lung carcinoma (NSCLC n=16), colon (n=10), pancreatic (n=10) and ovarian (n=4) cancer patients (both tumor and adjacent non-tumor/normal) were collected from University of Texas Medical Branch (UTMB; Galveston) Cancer Center tissue bank. All fresh tissues were collected in accordance with institutions review board approval. Samples included resected ovary, endometrium, and fallopian tubes from healthy individuals were provided by our collaborators Drs. Qiu and Patel from the Department of Pathology, UTMB. Peripheral blood mono-nuclear (PBMN) cells were collected from healthy donors at UNMC. Lysate preparation began with approximately 100 mg of tissue washed in phosphate buffered saline (PBS) pH 7.4. Each tissue was minced into fine pieces and homogenized using a glass dounce homogenizer in 1 ml of cold lysis buffer containing 50 mM Tris-HCl pH 7.5, 150 mM NaCl, 1% Triton X-100, 0.1 mM EDTA and protease inhibitor cocktail buffer tablet (PI; Roche Diagnostics). Lysates were centrifuged at 14,000 rpm for 20 min at 4°C and the supernatants were stored at −80°C. Tissue extracts equivalent to 40-60 μg of total protein were separated by SDS/PAGE and subjected to Western blot analysis with different α-APE1 antibodies (Abs): mouse monoclonal Ab (1:2000 dilution, Novus Biologicals; # NB100-116); rabbit polyclonal Ab (1:300 dilution) raised against the whole protein [44]; N-terminal 1-14 aa specific goat polyclonal Ab (1:200 dilution, Novus Biologicals; # NB100-897). α-NEIL1 Ab at 1:1000 dilution [45], α-PCNA Ab at 1:1000 dilution (Calbiochem; # NA03), α-HSC70 at1: 5000 dilution (B6-Sc7298, Santa Cruz Biotechnology), α-α-Tubulin at 1: 2000 dilution (Sigma: # T6199) Abs were used. The affinity-purified AcAPE1 Ab (1:200 dilution) was raised against the N-terminal 1-13 aa containing peptide with acetylated Lys6 residue as described earlier [9, 11]. Tissue lysates prepared in the absence of protease inhibitors were used in the experiments for analyzing the effect of cellular protease(s) on recombinant APE1.

### *In vitro* cleavage assay

Recombinant APE1 was purified as described previously [46]. APE1 protein (1 microgram) was incubated for 45 min at 37°C with extracts isolated from tumor or adjacent non-tumor tissue in the presence or absence of protease inhibitors. Western blot analysis was performed with different α-APE1 Abs on 100 nanograms of APE1. For dose and time course-experiments, 6 μg of APE1 was incubated with either increasing amounts (100 ng-20 μg) of tissue extracts for 45 min or with 10 μg of tissue extracts for different time periods in the absence of protease inhibitors and subjected to SDS/PAGE followed by Coomassie Blue staining.

### Cell lines, plasmids, siRNAs, transfection and treatments

Normal lung fibroblast IMR-90 (ATCC #CCL-186), human bronchial epithelial BEAS-2B (ATCC # CRL-9609) and lung adenocarcinoma A549 (ATCC # CCL-185) cell lines were cultured in DMEM/F12 1:1 or DMEM-high glucose (Gibco-BRL), with 10% fetal calf serum (FCS; Sigma), 100 U/ml penicillin and 100 μg/ml streptomycin (Gibco-BRL). hTERT-immortalized human foreskin fibroblast BJ-5ta (ATCC #CR-4001) cells were cultured in DMEM-low glucose medium (Gibco-BRL) with FCS and antibiotics. Human embryonic kidney HEK-293 (ATCC # CRL-1573) and inducible APE1-downregulated ^APE1siRNA^HEK-293T cells were cultured in DMEM-high glucose medium with FCS and antibiotics as described previously [11]. Human Colon cancer HCT116 (ATCC #CCL-247) were grown in MaCoy 5A medium. All cell lines were authenticated by STR DNA profiling by Genetica DNA laboratories, Burlington, NC, on August, 2015.

For knockdown experiments, exponentially growing cells at ≤ 40%-confluent were transfected with various doses of Dharmacon ON-TARGETplus APE1siRNA-J-010237-07 (siRNA1), APE1siRNA-J-010237-08 (siRNA3), APE1-siRNA2 (Sigma; ID: SASI_Hs01_00027147,) and universal control (Sigma) using Lipofectamine 2000 (Invitrogen) following manufacturer's protocol. Cells were harvested after 72 hrs. and total RNA was isolated before or after 4 hour treatment with TSA (100 ng/ml; Calbiochem). Generation of Wild-type (WT) APE1, N-terminal 42 amino acid deleted (NΔ42), mutant APE1 expression constructs in a pcDNA3 vector backbone and with FLAG-tagged WT APE1, K6R/K7R (RR), or N-terminal 33 amino acid deleted (NΔ33) mutant APE1 in PCMV5.1 expression plasmid was described earlier [8–11]. Single or combination mutations of residues K6,7,27,31,32 to glutamine (K5Q) or arginine (K5R) in FLAG-tagged WT APE1 were generated using a site-directed mutagenesis Kit (Stratagene) following manufacturer's protocol. Cells were transfected using Lipofectamine 2000 followed by protein and RNA isolation after 48 hrs.

### Cell cycle analysis

BJ-hTERT cells were synchronized by serum starvation in medium containing 0.05% serum for 72 hrs. Cells at G_o_/G1 stage were then supplemented with serum to allow cell cycle progression and cell lysates were prepared at different time points. Cell cycle studies were performed as previously described by FACS analysis at UTMB's Flow Cytometry Core Facility [11].

### Immunostaining

Overnight cultured cells on cover slips were fixed for 10 min at room temperature with solution containing 4% paraformaldehyde, 10 mM PIPES buffer pH 6.8, 2 mM EGTA, 2 mM MgCl_2_, 7% sucrose, 100 mM KCl, 50 mM NaF, and 10 mM sodium pyrophosphate. Cells were rinsed in PBS for 20 min, followed by permeabilization with 0.5% Triton X-100 in PBS, and then re-rinsed. Cells were incubated in blocking solution (3% bovine serum albumin in PBS) for 30 min followed by 1 hour incubation with primary Abs. Rabbit α-AcAPE1 (1:50) and mouse α-APE1 (1:100), in blocking solution at room temperature were used. After three 10 minute washes in PBS, cells were incubated with Alexa-Fluor 488, 568 and 633-conjugated secondary Abs in blocking solution (Molecular Probes/Invitrogen). Finally, cells were washed with PBS, counter stained with DAPI Ab (Invitrogen; 1:1000), and mounted. Images were obtained using a Zeiss LSM-510 confocal microscope with a 63X, 1.4 numerical aperture oil immersion objective at Confocal Imaging Laboratory at UNMC.

### Micro-array based GeneChip analysis

RNA from control and experimental samples in three biological replicates was submitted to UTMB Molecular Genomics Core Facility for carrying out micro-array based gene chip hybridization. An Affymetrix HGU133 Plus 2.0 array comprised of more than 54000 probe sets that corresponds to approximately 30,000 characterized genes was used. Data analysis was performed in Bioconductor/R using the CEL files. The R module/package Affy was used to perform the pre-processing steps which includes the RMA method for background correction and quantile normalization. Linear Models for MicroArray (Limma) was used to fit linear models for analyzing designed experiments and the assessment of overall gene expression and contrast analysis comparing the experimental groups [47]. The significance of a gene is calculated using the eBayes function that computes moderated F-statistics combing the t-statistics from all the available contrasts. This F-statistics determines if genes are differentially expressed across any contrast. The p-value is calculated based on the moderated t-statistics and F-statistics followed by FDR adjustment. Further, the modules topTable and decideTests were used to extract and summarize the results. To estimate overlap between two analyses, Venn Diagrams were created using the method “global” in the function “decideTests”. Heat maps were created on genes significant at p value < 0.001 or 0.00001. Finally the probe sets with fold change ≥ 1.25 (for up-regulated genes) and ≤ 0.8 (for down-regulated genes) were selected and uploaded to Ingenuity Systems based Pathway Analysis (IPA, Redwood City, California) for functional analysis. All microarray data have been submitted to NCBI following the guidelines for Minimum Information About a Microarray Gene Experiment (MIAME) and the accession number is GSE74572.

### Real time reverse transcription (RT)-PCR assay

Total RNA from control and experimental cells was isolated with the Qiagen RNeasy mini kit (Invitrogen). cDNA was synthesized using Superscript III first-strand synthesis kit (Invitrogen) following manufacturer's protocol. Expression level of the genes selected from micro-array analysis was analyzed by SYBR GREEN-based Real Time PCR with primers (RealTimePrimers.com) using HPRT1 as internal control. Data were represented as relative quantitation with respect to the reference samples set at 1 based on 2^−ΔΔCT^ method.

### Colony forming assay

APE1 siRNA-expressing HEK-293T (^APE1siRNA^HEK-293T) cells [11] were treated with doxycycline (Sigma; 1 μg/ml) for 5-6 days to knockdown endogenous APE1 and then transfected with WT or different mutant APE1 expression plasmids. Approximately 500 cells were plated on 60-mm dishes and allowed to grow for two weeks until visible colonies appear. The colonies were fixed with 100% methanol, stained with Giemsa staining solution (1:50) and counted.

## SUPPLEMENTARY FIGURES AND TABLES










