# Supplementary material for: Regulation of limited N-terminal proteolysis of APE1 in tumor via acetylation and its role in cell proliferation
Source: Oncotarget. 2016 Mar 10;7(16):22590–604. doi: 10.18632/oncotarget.8026 (PMC5008384; doi:10.18632/oncotarget.8026)
Supplement: Supplementary file 1 [file oncotarget-07-22590-s001.pdf]

SUPPLEMENTARY FIGURES AND TABLES

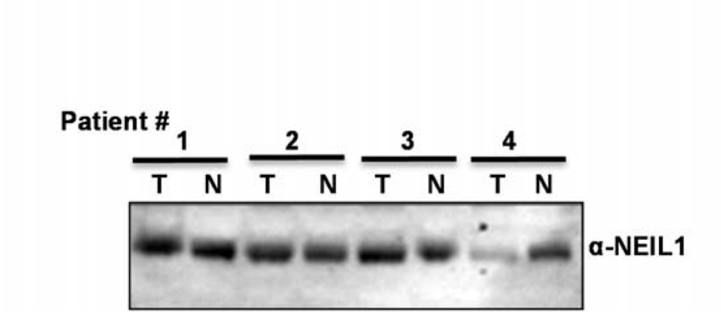

Supplementary Figure S1: Western blot analysis of tumor-adjacent non-tumor (N) and tumor (T) tissue extracts from NSCLC patients with α -NEIL1 Ab.

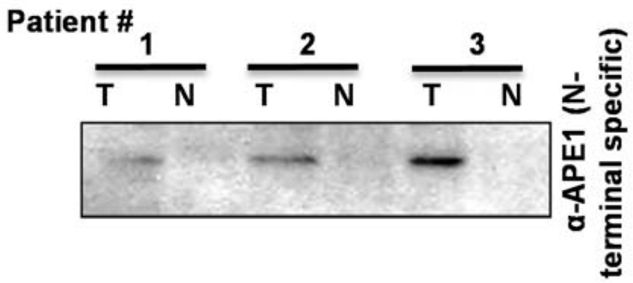

Supplementary Figure S2: Western blot analysis of tumor (T) and non-tumor (N) tissue extracts from colon cancer patients with N-terminal specific α -APE1 Ab.

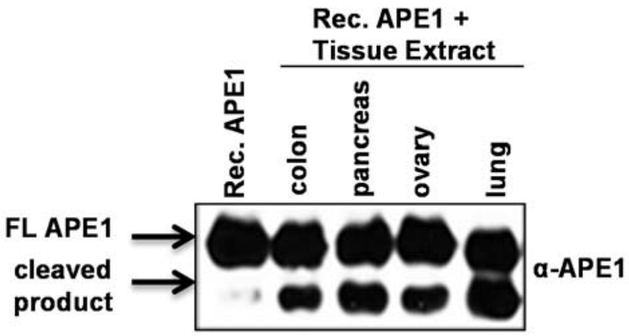

Supplementary Figure S3: Recombinant APE1 was incubated with diverse tumor-adjacent normal tissue extracts (isolated in the absence of protease inhibitors), and then immuno-blotted with α -APE1 Ab.

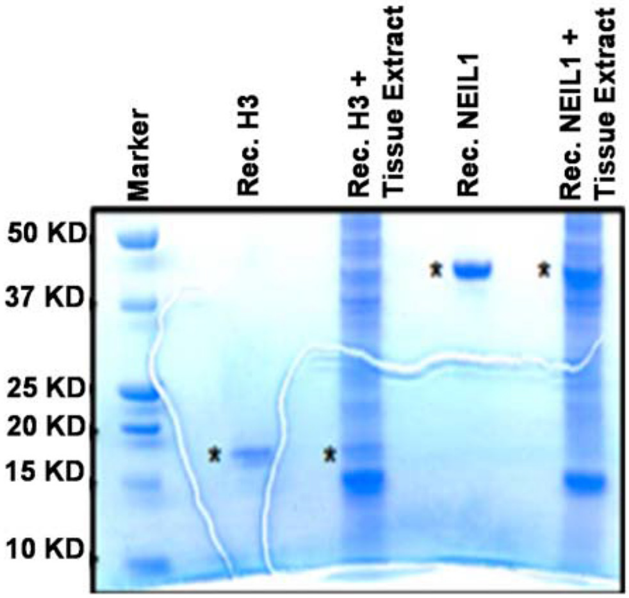

Supplementary Figure S4: Recombinant histone H3 (2 µg) or NEIL1 (6 µg) was incubated (45 min at 37°C) with tissue extract, separated in SDS/PAGE and visualized with Coomassie Blue staining. Rec: recombinant, FL: full length. * denotes the position of the corresponding recombinant protein's band.

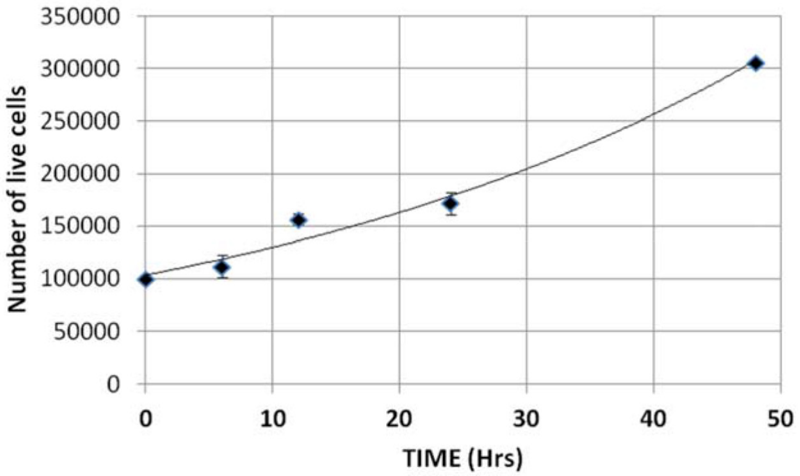

Supplementary Figure S5: 10,000 BJ-hTERT transformed cells were seeded in each plates. Cells were harvested at indicated time points after seeding and live cells were counted. Data from three independent experiments was plotted and a regression curve of best fit applied.

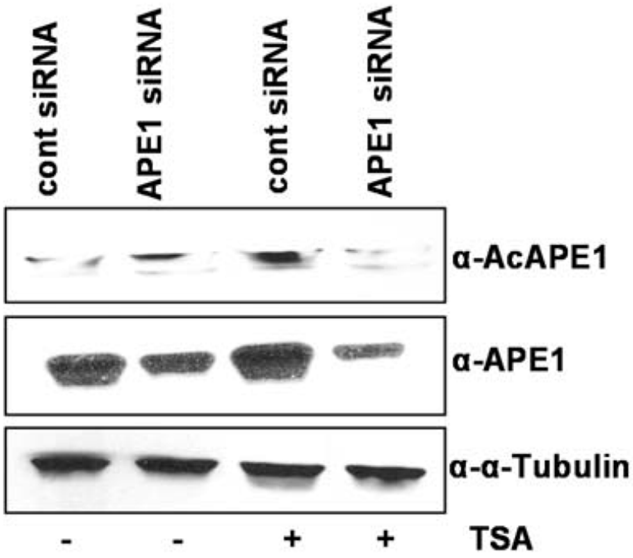

Supplementary Figure S6: Western blot analysis of AcAPE1, APE1 and α -Tubulin levels in control and APE1 knockdown cells (by siRNA transfection), followed by TSA (100 ng/ml, 4 hrs)-treatment.

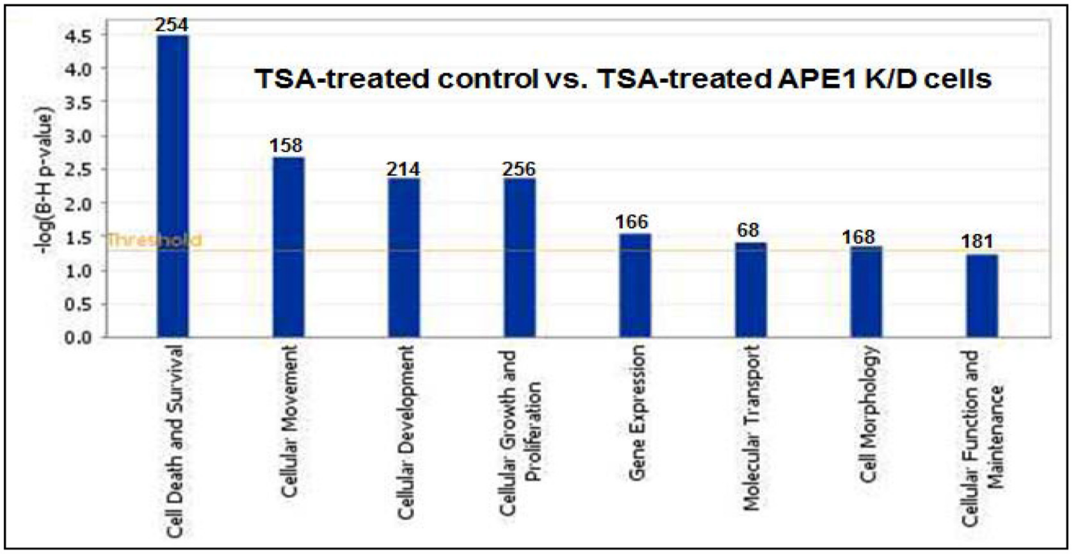

Supplementary Figure S7: IPA-based functional analysis of the genes with respect to cellular functions. Functional categories e.g. cellular functions are displayed along the x-axis and the −log(Benjamini-Hochberg p-value) are displayed along the y-axis; the horizontal line denotes the cut-off for significance (p-value of 0.05). The values above each bar denote the number of genes in particular category.

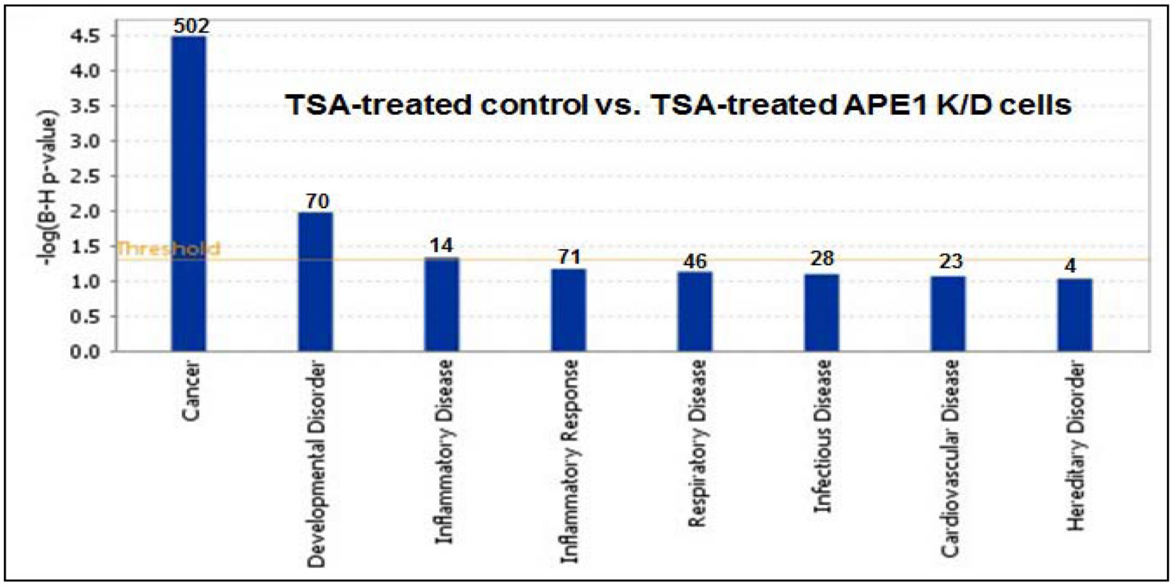

Supplementary Figure S8: IPA-based functional analysis of the genes with respect to diseases. Functional categories e.g. diseases are displayed along the x-axis and the −log(Benjamini-Hochberg p-value) are displayed along the y-axis; the horizontal line denotes the cut-off for significance (p-value of 0.05). The values above each bar denote the number of genes in particular category.

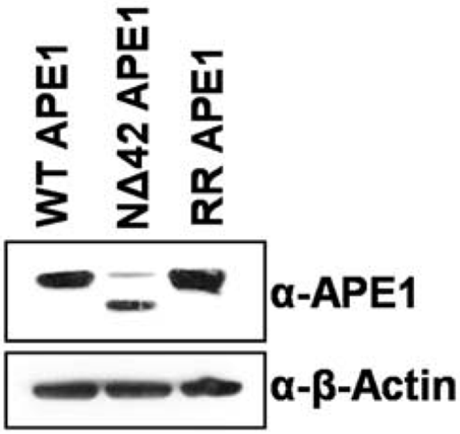

Supplementary Figure S9: Western blot analysis of APE1 and α -Tubulin levels in ectopic WT, NΔ42, RR APE1 expressing BEAS-2B cells.

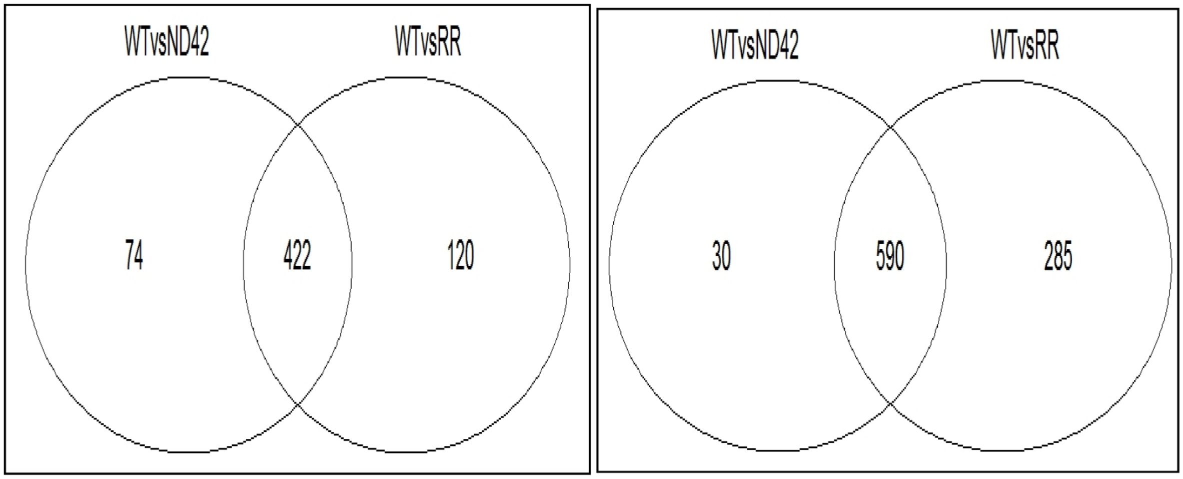

Supplementary Figure S10: Venn diagram (Limma; Decide Test method: global; p =0.0001) shows overlap of up- and down-regulated genes between ectopic WT vs. NΔ42 and WT vs. RR expressing BEAS-2B cells.

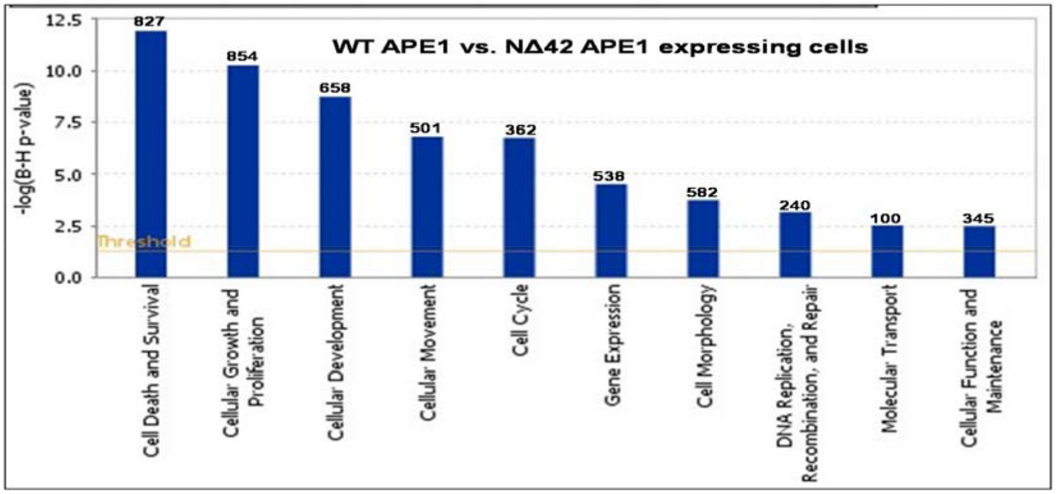

Supplementary Figure S11: IPA-based functional analysis of the genes with respect to cellular functions as described in Figure S7.

Supplementary Table 1: control vs. APE1 siRNA

See Supplementary File 1

Supplementary Table 2: TSA-treated control vs. TSA-treated APE1 siRNA

See Supplementary File 2

Supplementary Table 3: WT vs. NΔ42 APE1

See Supplementary File 3

Supplementary Table 4: WT vs. K6R/K7R (RR) APE1

See Supplementary File 4
